# Real-World Experience of the Association of HLADQA1*05 Allele With Loss of Response to Anti-TNF Inhibitors

**DOI:** 10.1093/crocol/otae058

**Published:** 2024-10-23

**Authors:** Aastha Chokshi, Christina A Raker, Sean Fine

**Affiliations:** Department of Internal Medicine, Warren Alpert Medical School of Brown University, Providence, RI, USA; Department of Biostatistics Lifespan, Epidemiology, Research Design, and Informatics Core, Providence, RI, USA; Division of Gastroenterology, Department of Medicine, Warren Alpert Medical School of Brown University, Providence, RI, USA

**Keywords:** anti-TNF, HLADQA1*05, immunogenicity

## Abstract

**Background:**

Antitumor necrosis factor (anti-TNF) biologics have revolutionized the treatment of inflammatory bowel disease (IBD). Previously, studies have shown an association between the HLADQA1*05 allele and the development of antibodies and were predictive of loss of response. We sought to investigate the rate of the HLADQA1*05 allele in patients with IBD at a New England center and its association with antibody development and discontinuation of anti-TNF therapy.

**Methods:**

A single center retrospective cohort study with patients on anti-TNF inhibitor therapy being followed at our IBD clinic who had testing performed for the HLADQA1*05 allele were identified and separated into 2 different groups: HLADQA1*05 positive (HLA carriers) or HLADQA1*05 negative (HLA noncarriers). Persistence of remaining on anti-TNF therapy, measurement of drug/antibody levels, and need for dose escalation were collected and stratified amongst the 2 groups.

**Results:**

The prevalence of the HLADQA1*05 allele among all IBD patients followed was 53%. We identified 67 IBD patients being treated with anti-TNF medications, 46 (69%) patients with Crohn’s disease and 21 (31%) with ulcerative colitis. Most of the HLA carriers (85%) and HLA noncarriers (92%) remained on anti-TNF therapy at the end of the study period. Thirty-six (84%) patients had therapeutic drug monitoring performed during maintenance therapy. Three patients in the HLA carrier group had meaningful antidrug antibody levels necessitating cessation of therapy compared to one patient in the HLA noncarrier group (*P* = .61). Only 3 (13%) of HLA carriers and 4 (21%) of HLA noncarriers were on combination therapy with an immunomodulator. 65% of HLA carriers required dose escalation compared to 50% of HLA noncarriers (*P* = .70).

**Conclusions:**

The prevalence of the HLADQA1*05 allele was 53% in our New England IBD patient population, similar to what has previously been reported in European studies. The majority of patients remained on anti-TNF therapy at the end of the study period despite carrier status. While there was a trend toward increased need for dose escalation among HLA carriers, this was not statistically significant. Future studies are needed to determine if the presence of the HLADQA1*05 allele leads to antibody development against anti-TNF inhibitors and treatment failure in patients with IBD.

## Graphical Abstract

**Graphical Abstract:**
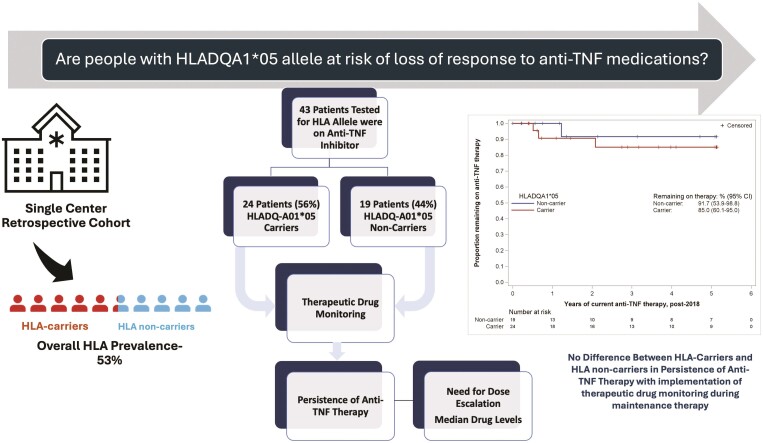

## Introduction

Antitumor necrosis factor (anti-TNF) agents have revolutionized the care for patients with inflammatory bowel disease (IBD) and are commonly prescribed biologics. However, both primary and secondary loss of response (SLR) remains a major concern.^1^ Primary nonresponse is the lack of improvement in the symptoms with induction therapy, and its incidence varies between 13% and 30% in clinical practice.^2^ Secondary loss of response describes an initial response to induction therapy but subsequent loss of response during maintenance therapy.^2^ The incidence of SLR is variable, though prior studies have shown an annual rate of 13% per patient-year of treatment for infliximab and 20.3% per patient-year of treatment for adalimumab.^2–4^ Immunogenicity through the development of antidrug antibodies (ADA) is one of the most common reasons for loss of response to anti-TNF medications.^5^ ADAs may lead to hypersensitivity reactions in patients and/or inhibit drug function, and studies have demonstrated ADAs correlate with both loss of response and infusion reactions.^5^ There are several strategies which have been employed to help reduce the risk of immunogenicity. Combination therapy with an immunomodulator (methotrexate, 6-mercaptopurine, or azathioprine) has been shown to help reduce the risk of immunogenicity and achieve higher remission rates than anti-TNF inhibitor monotherapy.^6–8^ Therapeutic drug monitoring (TDM) can measure both serum drug concentrations as well as ADAs and can provide practitioners with objective data for optimizing treatment. However, proactive TDM monitoring is not a widely accepted practice of care for patients with IBD. Therefore, when monitoring is performed reactively in response to patients’ symptoms, ADAs may have already developed and limit further treatment options with the agent.

There remains great interest in personalized medicine in being able to identify patients who are at risk for the development of ADAs in order to offer the most effective upfront treatment strategy when starting therapy. A European study showed a genome-wide association between the human leukocyte antigen (HLA) gene region, HLADQA1*05, and loss of response to anti-TNF medications due to increased risk of developing ADA.^9^ This was confirmed by a study that also showed the HLADQA1*05 allele is associated with ADA formation against infliximab and loss of response and treatment discontinuation.^10^ However, a more recent study suggests that among patients who have proactive TDM, the HLADQA1*05 allele was not associated with an increased need for treatment cessation or worse clinical outcomes.^11^

The majority of these genotype-associated outcome studies were conducted in a European patient population, and how these results translate to the United States patients remains uncertain. The goal of our study was twofold: (1) to determine the prevalence of the HLADQA1*05 allele among a New England IBD population, and (2) to investigate whether there exists an increased incidence of ADA development and treatment failure with anti-TNF inhibitor therapy in patients who are positive for the HLADQA1*05 allele.

## Methods

We conducted a single-center retrospective cohort study looking at patients with IBD followed at a New England IBD center between January 2018 and January 2023. The study protocol was approved by the Lifespan Health System Institutional Review Board (IRB# 1649439).

### Patients

Eligible subjects were 18 years of age or older with a diagnosis of moderate to severe Crohn’s Disease or Ulcerative Colitis. Data was collected on the patient’s demographics, disease type, disease extent, and severity. Patients’ medication history (including escalation of therapy), history of combination drug therapy with an immunomodulator, and TDM for drug and antibody levels were also collected. Our clinical practice tailored patient goal-directed care outcomes in a treat-to-target fashion that focused on a combination of clinical remission, laboratory normalization, and radiologic/and or endoscopic remission and was attributed to medication effectiveness/durability.

### HLADQA1*05 Assessment

Starting in 2020, as part of our center’s “pre-biologic” lab workup, testing included checking for the presence of the HLADQA1*05 allele. Patients who had previously been initiated on anti-TNF therapy prior to this time period (before 2020) were offered retrospective testing for the presence of the HLADQA1*05 allele. HLA genotype testing was performed using Quest Diagnostic lab (Chantilly, VA) and is a Polymerase Chain Reaction Amplification followed by Sequence Specific Oligonucleotide Probes to test for the HLADQA1 and HLADQA2 loci and was considered positive for our study if one of the alleles on the HLADQA1 loci was the HLADQA1*05 allele. The initial data analysis for determining the prevalence of the HLADQA1*05 allele was done using the entire IBD patient population. The remaining analyses were only performed for IBD patients on anti-TNF therapy. Patients who tested positive for the HLADQA1*05 allele while on anti-TNF therapy were grouped into the HLADQA1*05 carrier group (termed HLA carriers), and those who tested negative for the HLADQA1*05 allele were grouped into the HLADQA1*05 noncarrier group (termed HLA noncarriers).

### Therapeutic Drug Monitoring

Therapeutic drug monitoring performed proactively was the standard of care for our IBD clinic, and it was ordered at least once after the initial induction period of anti-TNF therapy in order to achieve desired drug concentration levels. Patients in the cohort had between 1 and 5 TDM levels performed postinduction, and completion was dependent on patient and infusion center compliance with performing and drawing labs. Serum sample analysis for TDM was performed by several different available laboratories during the study period and included Arup (electrochemiluminescence immunoassay, ECLIA), LabCorp (ECLIA), Prometheus (homogeneous mobility shift assay, HMSA), and Miraca laboratories (enzyme-linked immunosorbent assays, ELISA). Medication doses were dose optimized when TDM was followed through to achieve target trough drug levels, defined as being greater than 5 mcg/mL for infliximab and greater than 8 mcg/mL for adalimumab. If drug levels were not at target, dose escalation was pursued by either increasing the medication weight-based dosing and/or reducing intervals between infusions in order to achieve target drug concentrations on follow-up TDM testing. Given the potential difficulty in interpreting ADA levels between the different available drug assays, a “meaningful” ADA was defined in this study as immunogenic pharmacokinetic (PK) failure defined by low or undetectable drug concentrations and high ADA as reported by the specific assay. Patients with a “meaningful” ADA in our study only had one TDM performed as patients were deemed to have a nonresponse and had the drug discontinued. All data was collected using REDCap (Research Electronic Data Capture) software hosted at Lifespan Hospitals.

### Primary and Secondary Endpoints

The primary endpoint of our study was treatment persistence on anti-TNF therapy with either infliximab or adalimumab. Follow-up duration was determined from initiation of anti-TNF inhibitor. The secondary endpoint was needed for dose optimization. Standard dosing was defined as infliximab 5 mg/kg every 8 weeks and adalimumab 40 mg/kg every other week. Increased weight-based dosing or increased frequency was considered dose escalation. Finally, median drug levels were compared between HLA carriers and HLA noncarriers.

Patients who were hospitalized for an acute IBD flare and needed inpatient biologic initiation with an anti-TNF inhibitor were excluded from the dose escalation analysis as it was felt this patient population would initially be treated with escalated algorithms upfront.

### Statistical Analysis

Characteristics of the study population were summarized by descriptive statistics, and the prevalence of HLADQA1*05 was estimated along with an exact binomial 95% CI. Categorical variables were compared using chi-square or Fisher’s exact test, and continuous variables were compared using HLA carrier status using the *t*-test or Wilcoxon rank sum test. The duration of current anti-TNF therapy was examined by the Kaplan–Meier estimator. Drug levels and ADA levels were averaged across all results when more than one measurement was available. As a sensitivity analysis, drug levels were also examined from the final test performed. Two-tailed p-values were presented with *P* < .05 considered statistically significant. Data analysis was performed with SAS version 9.4 (SAS Institute).

## Results

### Patient Demographics

**Figure 1.**
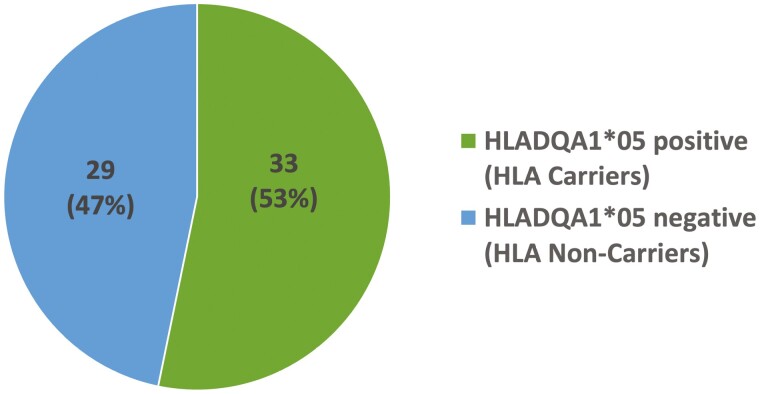
Patients tested for HLA allele and overall prevalence of HLADQ-A01*05 allele among all IBD patients.

During the study period, we reviewed 138 patients with IBD on biologic therapy, of which 62 had completed HLA testing and 33 patients were found to be carriers of the HLADQA1*05 Allele (53%, 95% CI, 40%-66%) (Figure 1). 67 of the 138 (49%) patients on biologic treatment were being managed with anti-TNF inhibitor therapy, 44 (67%) patients were on infliximab and 23 (34%) patients were on adalimumab. 46 (69%) patients had Crohn’s disease and 21 (31%) had ulcerative colitis (Table 1). The majority of patients were male (43/67, 64%), Caucasian (57/67, 85%) and only 7 (11%) patients were current smokers. Most of the patients on anti-TNF therapy (83%) had at least one or more TDM tests performed during the maintenance period.

**Table 1.** Characteristics of patients on anti-TNF therapy.

Total (*n*)	*N* = 67
Male, *n* (%)	43 (64)
Age at diagnosis (y)	
Median (Q1-Q3)	21 (16-30)
Min-Max	6-65
Duration of disease at chart review (y)	
Median (Q1-Q3)	6.9 (2.2-9.9)
Min-Max	0.19-36.9
Race, *n* (%)	
White	57 (85)
Black	4 (6)
Asian	2 (3)
Other	4 (6)
Smoking status, *n* (%)	
Never	48 (72)
Past	10 (15)
Current	7 (11)
Unknown	2 (3)
Crohn’s disease, *n* (%)	46 (69)
Phenotype, *n* (%)	
B1: Inflammatory	34 (74)
B2: Fibrostenotic	1 (2)
B3: Penetrating	11 (24)
Location^a^, *n* (%)	
L1: Terminal ileal	7 (15)
L2: Colonic	6 (13)
L3: Ileocolonic	33 (72)
L4: Upper gut	2 (4)
Perianal disease, *n* (%)	5 (11)
Ulcerative colitis, *n* (%)	21 (31)
Extent, *n* (%)	
E1: Proctitis	2 (10)
E2: Left-sided colitis	3 (14)
E3: Pancolitis	16 (76)
Anti-TNF agent, *n* (%)	
Infliximab	44 (66)
Adalimumab	23 (34)
History of intestinal surgery, *n* (%)	9 (13)
More than one prior surgery, *n* (%)	2 (22)

Abbreviation: TNF, tumor necrosis factor

^a^Categories were not mutually exclusive.

### HLA Carriers and HLA Noncarriers on Anti-TNF Therapy

**Figure 2.**
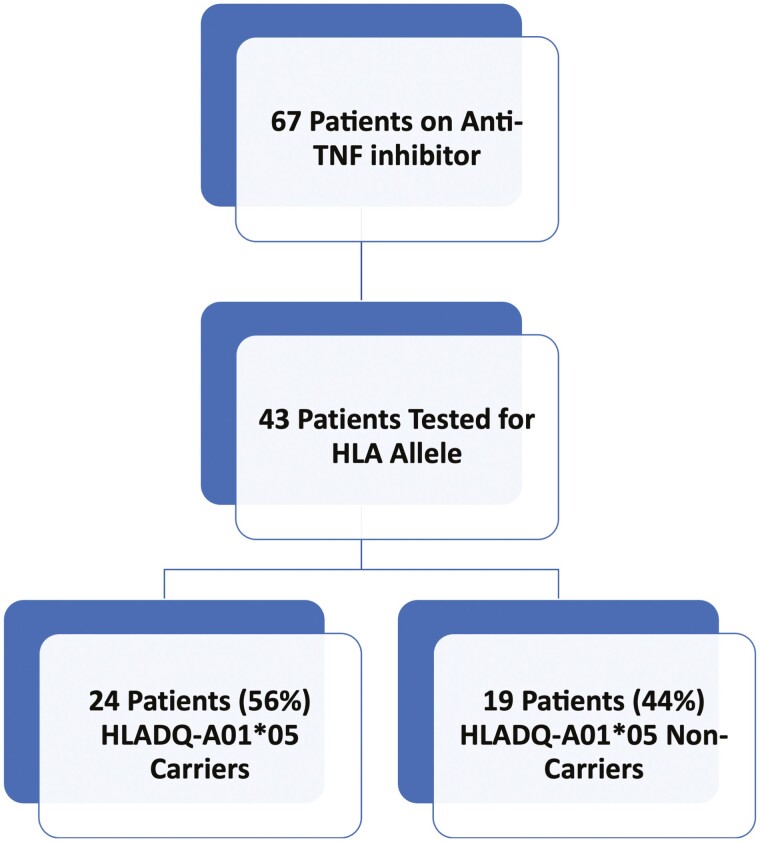
Patients tested for HLA allele among IBD patients on anti-TNF inhibitor therapy.

**Figure 3.**
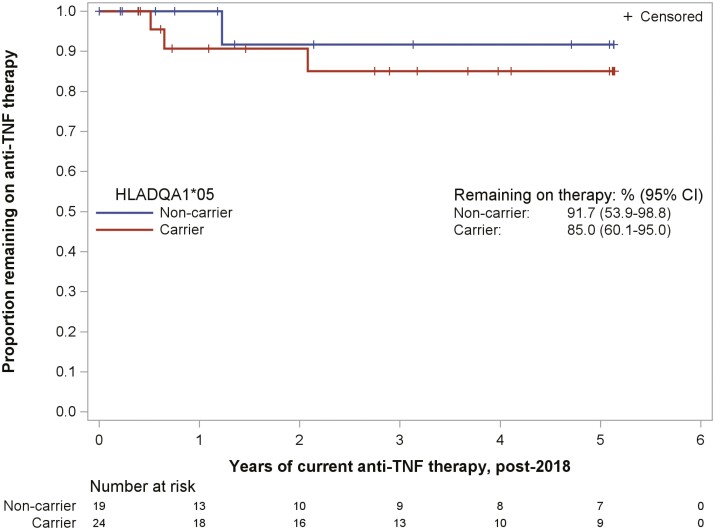
Anti-TNF therapy persistence by HLADQA1*05 carrier status.

Among the patients on anti-TNF therapy (*n* = 67), 43 (64%) patients completed testing for the HLA genotype (Figure 2). There was a total of 24 (56%) HLA carriers and 19 (44.2%) HLA noncarriers. There was no significant difference between gender, race, and disease type among HLA carriers and noncarriers (Table 2). Seventeen (71%) of the HLA carriers were on infliximab, compared to thirteen (68%) HLA noncarriers on infliximab (*P* = 1.00) and 7 (29%) HLA carriers were on adalimumab, compared to 6 (32%) HLA noncarriers on adalimumab (*P* = 1.00) (Table 2). Our primary endpoint was the persistence of anti-TNF therapy among HLA carriers and HLA noncarriers. A Kaplan–Meier analysis was used to determine the duration of current anti-TNF therapy, which showed that a majority of HLA carriers (85%) and noncarriers (92%) remained on anti-TNF therapy at the end of the study follow-up period (Figure 3).

**Table 2.** Characteristics of patients on anti-TNF therapy by HLADQA1*05 carrier status.

	HLA carrier	HLA noncarrier	*P*-value
Total, *n* (%)	24 (56)	19 (44)	
Male, *n* (%)	13 (54)	14 (74)	.22
Race, *n* (%)			.29
White	20 (83)	17 (90)	
Black	1 (4)	1 (5)	
Asian	0 (0)	1 (5)	
Other	3 (13)	0 (0)	
Disease type, *n* (%)			.10
Crohn’s disease	19 (79)	10 (53)	
Ulcerative colitis	5 (21)	9 (47)	
History of intestinal surgery, *n* (%)	2 (8)	2 (11)	1.00
Anti-TNF agent, *n* (%)			1.00
Infliximab	17 (71)	13 (68)	
Adalimumab	7 (29)	6 (32)	
Still on anti-TNF therapy, *n* (%)	21 (88)	18 (95)	.62
Currently on combination therapy, *n* (%)	3 (13)	4 (21)	.68
Dose escalation, if not hospitalized when treatment was initiated *n* (%)	13/20 (65)	8/16 (50)	.70

Abbreviations: HLA, human leukocyte antigen; TNF, tumor necrosis factor.

Our secondary endpoint included the need for dose escalation to achieve target trough levels as well as need for combination therapy. While we noted an increased need for dose escalation among the HLA carriers (13 patients, 65%) compared to the noncarrier group (8 patients, 50%), this was not statistically significant (*P* = .70) (Table 2). A small number of patients, 3 patients (13%) in the HLA carrier group and 4 patients (21%) in the HLA noncarrier group were on combination therapy with an immunomodulator (*P* = .68), but this resulted in no differences in outcomes.

Therapeutic drug monitoring was performed for 20 patients (83%) in the HLA carrier group and 16 patients (84%) in the noncarrier group (Table 3). Therapeutic drug monitoring was performed using several different laboratories, including Arup Laboratories (48%), LabCorp (42%), Prometheus (6%), and Miraca Labs (4%). There was a lower number of patients with adequate mean trough drug levels among HLA carriers (12 patients, 60%) compared to HLA noncarriers (14 patients, 88%), but this was not associated with treatment cessation (*P* = .13, Table 3). Overall, for all patients on infliximab, the median drug level among HLA carriers was 13.7 mcg/mL compared to 12.6 mcg/mL among HLA noncarriers (*P* = .77) (Table 3). For patients on adalimumab, the median drug level was lower in HLA carriers, 5.3 mcg/mL, compared to 10.0 mcg/mL among HLA noncarriers (*P* = .18) (Table 3). The results were similar when only the final test was analyzed (Table 3). Three patients in the HLA carrier group had high levels of ADA necessitating cessation of therapy compared to 1 patient in the HLA noncarrier group (*P* = .61) (Table 3).

**Table 3.** Drug levels and antibody levels by HLADQA1*05 carrier status.

	HLA carrier	HLA noncarrier	*P*-value
Total who received TDM	20	16	
Number of patients with average adequate drug level, *n* (%)	12 (60)	14 (88)	0.13
Number of patients with adequate drug level on final test, *n* (%)	10 (50)	11 (69)	0.32
Number of patients with a meaningful detected antibody, *n* (%)	3 (15)	1 (6)	0.61
Drug-specific levels			
Infliximab	(*n* = 13)	(*n* = 12)	
Median average drug level (mcg/mL)	13.7	12.6	0.77
Median drug level on final test (mcg/mL)	11.4	7.4	0.50
Adalimumab	(*n* = 7)	(*n* = 4)	
Median average drug level (mcg/mL)	5.3	10	0.18
Median drug level on final test (mcg/mL)	5.3	10	0.18

Abbreviation: TDM, therapeutic drug monitoring.

An adequate drug level was defined as being greater than 5 mcg/mL for infliximab and greater than 8 mcg/mL for adalimumab. Drug levels were analyzed 2 ways: by averaging across all available test results for each patient (range: 1-5 tests) and based on the final test performed.

## Discussion

We conducted a real-world single-center retrospective cohort study looking at 67 patients with IBD on anti-TNF inhibitor therapy being actively followed at a New England IBD center to determine the prevalence of the HLADQA1*05 allele and to investigate the association between HLA status with loss of response and immunogenicity to anti-TNF therapy. Identifying effective and patient-specific treatments through the use of precision medicine continues to be a goal in tailoring IBD treatment but thus far remains in its infancy. Recent findings in Europe have demonstrated an increased risk for loss of response and development of ADA to anti-TNF therapy among patients with the HLADQA1*05 allele. It is postulated that the HLA genetic variation may affect amino acids in the antigen-binding groove and that this ultimately has downstream effects on immune cell activation.^12^ The prevalence of the HLADQA1*05 allele was reported as approximately 40-46.4% in previous European studies.^9,11,13^ In our cohort, the prevalence rate of the HLADQA1*05 allele was 53% (95% CI, 40.1%-66.0%), similar to what has been observed. The PANTS trial showed that the HLADQA1*05 allele is associated with an increased risk of immunogenicity and loss of response with anti-TNF medications, with a hazard ratio of 1.90. The highest risk of immunogenicity at 1 year, 92%, was against infliximab among HLA carriers, whereas the lowest risk, 10%, was for patients on adalimumab who did not carry the allele.^9^ However, additional studies have shown that the presence of the HLA allele was not associated with increased rates of treatment cessation with anti-TNF inhibitors.^14^

Our results show that HLADQA1*05 allele carriers may not be at a higher risk of treatment cessation with anti-TNF inhibitors compared to noncarriers with the use of TDM in the maintenance period to optimized drug levels. In a single-center prospective Precision trial looking at a dashboard-guided dosing strategy for infliximab during induction, a post hoc analysis of the data demonstrated that the development of ADA and durability of infliximab were not dependent on HLA status but rather on low initial trough levels.^14^ The Precision dashboard trial used a strict proactive TDM model that included drug level, ADA level, weight, inflammatory markers, and albumin. Fuentes-Valenzuela et al. demonstrated in a study of 112 patients in Spain with TDM, at least 2 drug levels in the first year of treatment and 1 in the following years, that there was no impact on HLA status and drug persistence.^11^ In our study cohort, TDM was not performed during induction but rather during maintenance therapy and optimized as needed to achieve treatment threshold targets (greater than 5 mcg/mL for infliximab and greater than 8 mcg/mL for adalimumab). We purposefully excluded patients from the analysis within our cohort if they were hospitalized and treated with an accelerated dosing regimen of anti-TNF to avoid the possible effect of high drug concentrations on HLA carrier status outcomes. While there was a trend towards lower number of patients among HLA carriers with adequate trough levels during maintenance testing, 60% among HLA carriers versus 88% among HLA noncarriers, respectively, there was no impact on the durability of treatment on follow-up. There was no significant difference in median drug concentration levels during the maintenance period in the HLA carrier and noncarrier groups treated with infliximab and adalimumab, respectively.

Negating the development of ADA in patients treated with anti-TNF therapy remains crucial to avoid SLR treatment. Combination therapy with an immunomodulator has been reported to drastically lessen the risk for ADA development and improve treatment outcomes, as was seen in the SONIC trial.^6^ However, the use of immunomodulators has been associated with an increased risk of infections, specifically opportunistic, as well as a higher risk of nonmelanoma skin cancer and lymphomas.^15–17^ Importantly, a post hoc analysis of the SONIC trial demonstrated that there was no difference between infliximab monotherapy and higher quartile drug levels compared to combination therapy.^18^ In our present study, we had a very small percentage of patients on combination therapy, and there was no difference in the need for treatment cessation or antibody development between the HLA carrier and noncarrier groups. Only 3 patients in the HLA carrier group and 1 patient in the HLA noncarrier group had meaningful ADA antibodies necessitating cessation of medication, but this was not statistically significant between the groups.

Our study has limitations that need to be considered. The retrospective nature of our study certainly limits the ability to follow patients forward after initiation of therapy and monitor for antibody development, drug levels, and loss of response. Our patient cohort was smaller compared to the study done in Europe in a similar manner looking retrospectively, about half the size. Furthermore, there was a lack of diverse ethnic backgrounds in our study, which was largely Caucasian, which may make it difficult to extrapolate the findings toward the broader populations of patients with IBD in the United States.^11^ Another limitation of our study was the variability of lab assays that were utilized to detect drug and antibody levels, and it may be difficult to compare absolute concentrations between assays. Studies have demonstrated that there may be discrepancies when measuring biological drug concentrations as well as antibody levels among various assays.^19^ We did, however, try to limit any difficulty in interpreting the presence of meaningful ADAs (neutralizing antibodies) between the different assays by the strict definition as was described in the methods to avoid confusion with the different reporting units for antibody presence.

Our study strengths include that it reflects a real-world IBD practice and there was a substantial duration of time for a follow-up on anti-TNF therapy between the 2 groups. We suspect that the use of target drug optimization for anti-TNF therapy, as was performed in both the HLA carrier and noncarrier groups, was in part the reasoning as to why there was not a strong signal of a markedly higher rate of treatment failure in the HLA carrier group as has been previously reported.^11^ Our study findings are important because previously in the PANTS study it was hypothesized that for patients who are HLA carriers in whom immunomodulators are contraindicated or not tolerated, practitioners may want to advise against the use of anti-TNF drugs.^9^ Based on our results, while HLA carriers may have had a higher need for dose escalation to achieve target trough levels, this did not translate into higher rates of antibody development or treatment cessation. The lower end of accepted levels for both infliximab and adalimumab based on expert opinion, are 5 mcg/ml and 8 mcg/ml, respectively.^19,20^ It is possible that the reason we did not see any impact of HLA carrier status on anti-TNF therapy in our study was that the goal was to optimize the dose of patients to these levels. Ongoing studies with a larger sample size can help further elucidate the role of the HLA genotype and its impact on immunogenicity with anti-TNF inhibitors in IBD patients.

In summary, our study highlights that the presence of the HLADQA1*05 allele in patients with IBD who live in the United States is similar to what has previously been reported in European studies. HLA carriers, compared to noncarriers treated with anti-TNF therapy, were not at risk for higher rates of treatment cessation or development of ADAs. Therapeutic drug monitoring may overcome the concern of HLA carriers and help assess the need for dose escalation to prevent SLR. Future studies for patients treated with anti-TNF therapy need to continue to address the HLADQA1*05 genotype and its impact on outcomes.

## Data Availability

Data are not publicly available.
